# Circulating Interleukin-17 Across Airway Disease Phenotypes: A Single-Center Observational Study

**DOI:** 10.3390/jcm15187003

**Published:** 2026-09-10

**Authors:** Corina Porr, Valentin-Cristian Iovin, Anca Vidrighin, Emi M. Preda, Gabriela Mariana Iancu, Dana M. Harris, Cosmina Diaconu

**Affiliations:** 1 Allergology Department, Faculty of Medicine, Lucian Blaga University of Sibiu, 550169 Sibiu, Romania; corina.porr@ulbsibiu.ro; 2 Department of Allergology, County Clinical Emergency Hospital Sibiu, Bulevardul Corneliu Coposu 2–4, 550245 Sibiu, Romania; cosmina.diaconu@ulbsibiu.ro; 3 Doctoral School Department, “Victor Babes” University of Medicine and Pharmacy, 300041 Timisoara, Romania; valentin.iovin@umft.ro; 4 Department III Functional Sciences, Physiology Discipline, “Victor Babes” University of Medicine and Pharmacy, 300041 Timisoara, Romania; 5 Centre of Immuno-Physiology and Biotechnologies (CIFBIOTEH), Department of Functional Sciences, Physiology, “Victor Babes” University of Medicine and Pharmacy, 300041 Timisoara, Romania; 6 Department of Paediatrics, Faculty of Medicine, Lucian Blaga University of Sibiu, 550169 Sibiu, Romania; 7 Department of Radiology, “Carol Davila” University of Medicine and Pharmacy, 050474 Bucharest, Romania; emi.preda@umfcd.ro; 8 Department of Radiology and Medical Imaging, “Foișor” Clinical Hospital of Orthopaedics, Traumatology and Osteoarticular Tuberculosis, 021382 Bucharest, Romania; 9 Department of Dermatology, Faculty of Medicine, Lucian Blaga University of Sibiu, 550169 Sibiu, Romania; 10 Internal Medicine Department, Mayo Clinic, Jacksonville, FL 32224, USA; harris.dana@mayo.edu; 11 Department of Nursing and Dental Medicine, Faculty of Medicine, Lucian Blaga University of Sibiu, 550024 Sibiu, Romania

**Keywords:** interleukin-17, allergic rhinitis, asthma, airway inflammation, biomarkers, Th17 immunity, united airways, inflammatory endotyping

## Abstract

**Background:** Interleukin-17 (IL-17) contributes to persistent airway inflammation, neutrophil recruitment, and inflammatory pathways extending beyond classical type 2 immunity. However, the distribution of circulating IL-17 across clinically distinct upper- and lower-airway disease phenotypes remains incompletely characterized. This study compared serum IL-17 concentrations in allergic rhinitis, non-allergic asthma, allergic asthma associated with allergic rhinitis, and healthy controls. **Methods:** This retrospective single-center observational study included 88 adults: allergic rhinitis (*n* = 31), non-allergic asthma (*n* = 15), allergic asthma associated with allergic rhinitis (*n* = 22), and healthy controls (*n* = 20). Serum IL-17 concentrations were measured using a quantitative sandwich enzyme-linked immunosorbent assay. Overall group differences were assessed using the Kruskal–Wallis test, followed by Holm-adjusted pairwise Mann–Whitney U tests. **Results:** Serum IL-17 concentrations differed significantly across the four groups (Kruskal–Wallis H = 12.981, *p* = 0.0047). Median IL-17 concentrations were 126.21 pg/mL in non-allergic asthma, 50.97 pg/mL in allergic rhinitis, 54.72 pg/mL in allergic asthma associated with allergic rhinitis, and 0.00 pg/mL in healthy controls. Each disease group had significantly higher IL-17 than controls after Holm correction (adjusted *p* = 0.0103, 0.0172, and 0.0196, respectively), whereas the three disease phenotypes did not differ significantly from one another. **Conclusions:** Circulating IL-17 concentrations were higher in allergic rhinitis, non-allergic asthma, and allergic asthma associated with allergic rhinitis than in healthy controls. However, the substantial overlap among disease phenotypes and the absence of statistically significant between-phenotype differences should not be interpreted as evidence of equivalence or a shared biological mechanism. These findings should be considered exploratory and require confirmation in larger, prospectively characterized cohorts.

## 1. Introduction

Allergic airway diseases, including allergic rhinitis and asthma, are among the most prevalent chronic inflammatory disorders worldwide, affecting hundreds of millions of individuals and imposing a substantial burden on healthcare systems and quality of life [1,2,3]. Although traditionally considered distinct clinical entities, allergic rhinitis and asthma are now recognized as manifestations of a unified airway disease in which inflammation extends throughout the respiratory tract [2,4,5,6,7,8]. This concept is supported by strong epidemiological, anatomical, and immunological evidence demonstrating that upper and lower airway inflammation frequently coexist and mutually influence disease severity, symptom control, and long-term clinical outcomes [2,4,5,6,7,8].

Despite major advances in the understanding and treatment of allergic airway diseases, substantial clinical heterogeneity remains unexplained [9,10,11,12,13]. Patients sharing the same clinical diagnosis often exhibit markedly different inflammatory profiles, disease severity, therapeutic responses, and rates of disease progression [9,10,11,12,13]. Current classification based primarily on clinical manifestations provides limited insight into the underlying biological mechanisms, emphasizing the need for biomarkers capable of identifying inflammatory endotypes that may improve disease stratification and support precision medicine approaches [9,10,11,12,13,14,15].

The pathophysiology of allergic airway diseases has traditionally been explained by type 2 (T2) immune responses characterized by allergen-specific IgE production, eosinophilic inflammation, mast-cell activation, and increased expression of interleukin (IL)-4, IL-5, and IL-13 [3,16,17,18,19]. These discoveries have led to the development of highly effective biologic therapies targeting T2 inflammation [16,17,18]. However, accumulating evidence indicates that the classical Th2 paradigm cannot fully explain persistent inflammation, airway remodeling, corticosteroid resistance, and severe disease observed in a substantial proportion of patients [12,13,16,17,18,20,21,22,23,24]. These observations suggest the involvement of additional immune pathways extending beyond classical type 2 inflammation [24,25,26,27,28].

Among non-type 2 inflammatory pathways, the IL-17/Th17 axis has emerged as a key regulator of chronic airway inflammation [25,26,27,28]. IL-17A is produced not only by Th17 lymphocytes but also by innate immune cells, including γδ T cells, innate lymphoid cells, neutrophils, and macrophages, thereby linking innate and adaptive immunity [25,26,27,28]. Through activation of epithelial cells, fibroblasts, endothelial cells, and airway smooth muscle cells, IL-17 promotes the production of pro-inflammatory cytokines, chemokines, matrix metalloproteinases, and granulocyte colony-stimulating factor [26,27,28], leading to neutrophilic inflammation, persistent immune activation, airway remodeling, and impaired corticosteroid responsiveness [21,24,29,30,31,32,33]. Increasing evidence further suggests that IL-17 and Th2 pathways frequently coexist, generating mixed inflammatory endotypes that may contribute to disease severity and treatment resistance [16,24,28,32].

Although numerous studies have investigated IL-17 in asthma, most have focused on isolated disease phenotypes, selected populations, or severe asthma [20,21,29,30,31,32,33,34,35,36]. Comparatively little is known about whether circulating IL-17 reflects inflammatory activity across the entire spectrum of allergic airway diseases, including allergic rhinitis, non-allergic asthma, and allergic asthma associated with allergic rhinitis [12,13,14,15]. Consequently, it remains unclear whether circulating IL-17 reflects phenotype-specific inflammatory activity or a shared systemic inflammatory signal across airway diseases [4,5,6,7,8,14,15].

We hypothesized that circulating IL-17 concentrations would differ between participants with airway disease and healthy individuals. Therefore, the aim of the present study was to compare serum IL-17 concentrations among adults with allergic rhinitis, non-allergic asthma, allergic asthma associated with allergic rhinitis, and healthy controls using a uniform clinical and laboratory protocol. By evaluating upper-airway, lower-airway, and combined disease within the same cohort, we sought to characterize the distribution of circulating IL-17 across clinically distinct airway phenotypes without assuming biological equivalence among groups.

## 2. Materials and Methods

### 2.1. Study Design and Participants

This retrospective single-center observational study included 88 adult participants evaluated at the Department of Allergology, County Clinical Emergency Hospital Sibiu, Romania, between January and December 2025. Participants were classified into four groups according to their clinical diagnosis: allergic rhinitis (AR), non-allergic asthma (NAA), allergic asthma associated with allergic rhinitis (AA + AR), and healthy controls. Clinical participants were identified consecutively from adult patients evaluated in the Allergology Department who fulfilled the predefined diagnostic and eligibility criteria. Healthy controls were selected from adults evaluated during the same period who had no documented diagnosis of asthma or allergic rhinitis and for whom sufficient clinical information and an archived serum sample were available.

The diagnosis of allergic rhinitis was established according to Allergic Rhinitis and its Impact on Asthma (ARIA) recommendations, based on a compatible clinical history and symptoms together with evidence of IgE-mediated sensitization demonstrated by positive skin prick testing to clinically relevant aeroallergens [1,2]. Asthma was diagnosed according to Global Initiative for Asthma (GINA) recommendations on the basis of compatible respiratory symptoms and pulmonary function assessment [3]. Allergic asthma associated with allergic rhinitis was defined by the coexistence of a GINA-based diagnosis of asthma and ARIA-based allergic rhinitis with positive skin prick testing to relevant aeroallergens. Non-allergic asthma was defined as GINA-based physician-diagnosed asthma in the absence of sensitization to the common aeroallergens included in the skin prick test panel. The non-allergic asthma group was defined on the basis of the absence of demonstrable aeroallergen sensitization and was not further classified as eosinophilic or neutrophilic asthma, because complete peripheral blood eosinophil counts and airway inflammatory cell profiles were not consistently available in the archived dataset. No blood inflammatory biomarker, including total IgE or peripheral blood eosinophil count, was used as a mandatory diagnostic criterion for group allocation.

The inclusion criteria were: (1) age ≥18 years; (2) an established diagnosis of allergic rhinitis, non-allergic asthma, or allergic asthma associated with allergic rhinitis according to the diagnostic definitions described above, or eligibility as a healthy control; (3) disease duration of at least 2 years for participants with airway disease; (4) availability of sufficient archived clinical information to confirm group assignment; and (5) availability of an archived serum sample for IL-17 measurement. Healthy controls had no documented diagnosis of asthma or allergic rhinitis and were included only when sufficient clinical information was available to support control-group classification.

The exclusion criteria were acute respiratory infection at the time of clinical evaluation, autoimmune disease, malignant disease, chronic inflammatory disease unrelated to allergy, and current systemic immunosuppressive therapy.

For allergic rhinitis, disease severity was classified according to ARIA recommendations into mild intermittent, moderate/severe intermittent, mild persistent, and moderate/severe persistent disease. For participants with asthma, the ordinal categories available in the archived dataset corresponded to GINA treatment steps 1–4 at the time of clinical assessment. These GINA treatment steps were used as an available clinical treatment-intensity classification and were not interpreted as an independent biomarker-based severity endotype.

Given the retrospective design and the exclusive use of fully anonymized clinical data and archived serum samples previously collected during routine clinical care, formal prospective ethics committee approval was not required according to applicable institutional procedures. No additional biological samples were collected and no study-specific interventions were performed. The study was conducted in accordance with the principles of the Declaration of Helsinki.

### 2.2. Clinical Assessment

Demographic and clinical characteristics were recorded for all participants, including sex, body mass index (BMI), smoking status, family history of allergic diseases, and place of residence. All participants included in the study were adults. Clinical diagnoses were established according to ARIA and GINA recommendations. For exploratory characterization of allergic participants, sensitization profiles were classified as seasonal, perennial, or mixed according to the aeroallergen sensitization categories recorded in the archived clinical dataset. The number of sensitization categories was also recorded for exploratory correlation with serum IL-17 concentrations.

### 2.3. Measurement of Serum IL-17

Serum samples used for IL-17 measurement had originally been collected from peripheral venous blood during routine clinical evaluation between January and December 2025 and were retrieved from the institutional laboratory archive. No additional blood samples were obtained specifically for the present study. After routine processing, serum was separated by centrifugation, aliquoted, and stored at −80 °C until retrospective IL-17 analysis. The archived samples were linked to the corresponding clinical data through anonymized study identifiers. Serum IL-17 concentrations were measured using a commercially available quantitative sandwich enzyme-linked immunosorbent assay (Quantikine^®^, R&D Systems, Minneapolis, MN, USA) according to the manufacturer’s instructions. The archived dataset contained the numerical IL-17 concentrations generated by the laboratory analysis, including values recorded as 0.00 pg/mL. Because the original assay documentation required to distinguish the analytical limit of detection (LOD) from the lower limit of quantification (LLOQ) was not available for this retrospective analysis, no post hoc reassignment or imputation of low or zero values was performed. The concentrations were analyzed as recorded in the source dataset.

### 2.4. Statistical Analysis

The archived individual-level dataset was reanalyzed using Python version 3.13.5 (Python Software Foundation, Wilmington, DE, USA), pandas version 2.2.3, SciPy version 1.17.0, and statsmodels version 0.14.6. Because serum IL-17 values were markedly right-skewed and included zero values, continuous data are reported as mean ± standard deviation (SD), median, interquartile range (IQR), and observed minimum–maximum range. Overall differences among the four study groups were assessed using the Kruskal–Wallis test. When the global test was significant, pairwise comparisons were performed using two-sided Mann–Whitney U tests with Holm adjustment for multiple testing. A conventional one-way analysis of variance was performed as a sensitivity analysis. Exploratory associations between the number of aeroallergen sensitization categories and serum IL-17 concentrations were also assessed using Spearman rank correlation in the overall allergic population and separately within the allergic rhinitis and allergic asthma associated with allergic rhinitis groups. Categorical variables are reported as frequencies and percentages. All tests were two-sided, and *p* < 0.05 was considered statistically significant. A multivariable model adjusted simultaneously for age, sex, obesity, and smoking was not fitted because the phenotype-specific sample sizes were too small to support a stable model without substantial risk of overfitting; therefore, these potential confounders were treated descriptively rather than as confirmatory adjusted covariates. During manuscript preparation, ChatGPT (OpenAI, GPT-5.6) was used solely for language editing and readability improvement. The authors reviewed and edited all AI-assisted content and take full responsibility for the final content of the manuscript.

## 3. Results

### 3.1. Study Population

A total of 88 participants were included in the study and classified into four groups according to their clinical diagnosis: allergic rhinitis (AR, *n* = 31), non-allergic asthma (NAA, *n* = 15), allergic asthma associated with allergic rhinitis (AA + AR, *n* = 22), and healthy controls (*n* = 20).

Baseline demographic and clinical characteristics of the study population are summarized in Table 1. The healthy control group had a higher proportion of women than the three disease groups, indicating a relevant baseline imbalance in sex distribution. The distribution of sex and the evaluated clinical characteristics reflected the predefined diagnostic classification, and all patients had an established disease duration of at least two years.

Among the 53 participants with allergic disease, 14 (26.4%) had seasonal sensitization only, 20 (37.7%) had perennial sensitization only, and 19 (35.8%) had mixed seasonal and perennial sensitization. In the allergic rhinitis group (*n* = 31), 14 (45.2%) had seasonal-only sensitization, 7 (22.6%) perennial-only sensitization, and 10 (32.3%) mixed sensitization. In the allergic asthma associated with allergic rhinitis group (*n* = 22), 13 (59.1%) had perennial-only sensitization and 9 (40.9%) had mixed sensitization.

Thirty-one allergic participants were sensitized to one aeroallergen category, 17 to two categories, and 5 to three categories. The number of sensitization categories was not significantly associated with circulating IL-17 concentrations in the overall allergic population (Spearman rho = 0.169, *p* = 0.227), allergic rhinitis subgroup (rho = 0.099, *p* = 0.598), or allergic asthma associated with the allergic rhinitis subgroup (rho = 0.286, *p* = 0.198). Among the 33 participants sensitized to pollen, 17 (51.5%) had serum sampling during spring–summer and 16 (48.5%) during autumn–winter. In the allergic rhinitis subgroup, 13 pollen-sensitized participants were sampled during spring–summer and 11 during autumn–winter; in the allergic asthma associated with allergic rhinitis subgroup, the corresponding numbers were 4 and 5. Because the archived dataset recorded broad seasonal periods rather than allergen-specific exposure windows, these data were interpreted descriptively and were not classified as definitive individual in-season versus off-season exposure.

### 3.2. Distribution of Serum IL-17 Across Clinical Phenotypes

Serum IL-17 concentrations were substantially higher in all three airway disease groups than in healthy controls. Detailed descriptive statistics are presented in Table 2. The overall group difference was statistically significant using the Kruskal–Wallis test (H = 12.981, *p* = 0.0047). A one-way ANOVA yielded a concordant global result (F = 5.349, *p* = 0.0020). Non-zero IL-17 concentrations were recorded in 11 of 15 participants with non-allergic asthma (73.3%), 20 of 31 participants with allergic rhinitis (64.5%), 14 of 22 participants with allergic asthma associated with allergic rhinitis (63.6%), and 8 of 20 healthy controls (40.0%).

Pairwise Mann–Whitney U tests with Holm correction showed significantly higher serum IL-17 in non-allergic asthma versus controls (adjusted *p* = 0.0103), allergic rhinitis versus controls (adjusted *p* = 0.0172), and allergic asthma associated with allergic rhinitis versus controls (adjusted *p* = 0.0196). No significant differences were observed among the three disease phenotypes (all Holm-adjusted *p* = 1.000).

### 3.3. Overall Pattern of Findings

The principal reproducible pattern was a marked elevation of circulating IL-17 in participants with airway disease compared with healthy controls, together with broadly overlapping distributions among allergic rhinitis, non-allergic asthma, and combined allergic airway disease. The lack of statistically significant differences among the three disease phenotypes should not be interpreted as evidence of equivalence, as the study was not designed or powered to establish equivalence between groups. The observed overlap may reflect either shared inflammatory features or insufficient statistical power to detect smaller between-phenotype differences.

## 4. Discussion

The present study demonstrated that circulating IL-17 concentrations were significantly higher in adults with allergic rhinitis, non-allergic asthma, and allergic asthma associated with allergic rhinitis than in healthy controls. In contrast, IL-17 distributions were broadly similar across the three clinical disease phenotypes. These findings support a difference between participants with airway disease and healthy controls but do not demonstrate clear discrimination among the three clinical phenotypes. Accordingly, the results should be interpreted as exploratory rather than confirmatory.

The observed elevation of circulating IL-17 is biologically plausible. IL-17 links innate and adaptive immune responses and activates epithelial cells, fibroblasts, endothelial cells, and airway smooth muscle cells [25,26,27,28]. Downstream effects include the release of pro-inflammatory cytokines, chemokines, granulocyte colony-stimulating factor, and matrix metalloproteinases, which promote neutrophil recruitment, persistent inflammation, and airway remodeling [21,24,26,27,28,30,31,32,33]. Airway remodeling in asthma also involves structural and vascular changes, including angiogenic pathways. Increased vascular endothelial growth factor expression has been associated with airway remodeling and irreversible bronchoconstriction in adult patients with asthma, further illustrating the complex interaction between inflammatory and structural mechanisms in chronic airway disease [37].

Our findings are consistent with previous reports demonstrating increased IL-17 expression or activity in asthma and other chronic inflammatory airway conditions [20,24,32,34,36,38]. Previous studies have linked the IL-17/Th17 pathway particularly to neutrophilic inflammation, airway hyperresponsiveness, corticosteroid resistance, and severe asthma [20,24,29,30,31,32,33,34,35,36]. This concept is further supported by dedicated reviews describing the role of IL-17 in severe asthma, steroid-insensitive inflammation, airway remodeling, and mixed inflammatory phenotypes, as well as by studies examining the interaction between the Th17/IL-17 axis and the airway microenvironment [39,40,41]. In the present cohort, circulating IL-17 concentrations were significantly higher in all three airway-disease groups than in healthy controls, but the distributions overlapped substantially among allergic rhinitis, non-allergic asthma, and allergic asthma associated with allergic rhinitis. Therefore, our data do not support a phenotype-specific interpretation of circulating IL-17. Rather, systemic IL-17 activity may occur across clinically distinct airway conditions, while its relationship with local airway inflammatory patterns and specific inflammatory endotypes remains unresolved. This distinction is particularly important because circulating cytokine concentrations cannot be assumed to directly reflect cellular or cytokine profiles within the airway compartment.

The presence of elevated circulating IL-17 in both upper- and lower-airway disease groups is compatible with the broader united-airways framework [2,4,5,6,7,8]. However, the present data do not establish a shared causal inflammatory mechanism across these phenotypes. Importantly, the present findings do not establish circulating IL-17 as a diagnostic, prognostic, or disease-specific biomarker. Rather, serum IL-17 should be interpreted here as an exploratory measure of systemic inflammatory activity.

The wide and overlapping IL-17 distributions observed within the disease groups indicate substantial biological heterogeneity. Several participants had zero or low recorded IL-17 concentrations, whereas others had markedly elevated concentrations. This variability supports the use of non-parametric analyses and suggests that IL-17 may be most informative as one component of a broader biomarker panel rather than as a stand-alone measure.

### 4.1. Clinical Implications

The clinical implications of these findings relate primarily to inflammatory endotyping. Current biologic therapies predominantly target type 2 pathways, while mixed and non-type 2 inflammation remains clinically relevant in subsets of patients [14,15,16,17,18,24,38]. Biological therapies targeting the IL-17/Th17 axis have also been investigated in asthma. Brodalumab, a monoclonal antibody targeting IL-17 receptor A, and secukinumab, which neutralizes IL-17A, have been evaluated in patients with moderate-to-severe or uncontrolled asthma [42,43]. However, clinical trials have not demonstrated consistent or clinically meaningful improvements in asthma control or lung function [42,43]. These findings indicate that, despite the biological relevance of the IL-17 pathway in airway inflammation, direct IL-17 blockade is not currently an established therapeutic strategy for asthma and may require more precise identification of responsive inflammatory endotypes. Circulating IL-17 may provide complementary information about systemic inflammatory activity, but prospective studies are required to determine whether it adds predictive value beyond established clinical and type 2 biomarkers.

### 4.2. Strengths and Limitations

The principal strength of the study is the simultaneous evaluation of three clinically distinct airway disease phenotypes and healthy controls using the same serum assay and a uniform single-center protocol. Reanalysis of the archived individual-level dataset also permitted transparent reporting of medians, interquartile ranges, observed ranges, and multiplicity-adjusted pairwise comparisons. In addition, the archived allergological data allowed an exploratory characterization of seasonal, perennial, and mixed sensitization patterns, as well as assessment of the number of aeroallergen sensitization categories in relation to circulating IL-17 concentrations.

Several limitations should be acknowledged. The study was retrospective, single-center, and included a modest sample size, particularly in the non-allergic asthma group. IL-17 was measured at a single time point, and circulating concentrations may not accurately reflect local airway inflammation. Induced sputum, bronchoalveolar lavage, cellular phenotyping, and additional cytokine measurements were not available. Complete blood count parameters, total serum IgE, and C-reactive protein were not consistently available across the entire archived cohort, preventing systematic comparison of IL-17 with conventional inflammatory and type 2 biomarkers. Accordingly, the non-allergic asthma subgroup could not be reliably further classified as eosinophilic or neutrophilic asthma, because peripheral blood eosinophil counts and airway inflammatory cell profiles were not consistently available in the archived dataset. The broad, zero-inflated IL-17 distributions limited the precision of subgroup analyses. A fully adjusted multivariable model incorporating age, sex, obesity, and smoking was not considered statistically stable because of the small phenotype-specific sample sizes and sparse subgroup cells. The marked difference in sex distribution between healthy controls and the disease groups represents an additional potential source of confounding. Because the available sample size did not support a stable multivariable adjustment, residual confounding by sex and other baseline characteristics cannot be excluded.

Although sensitization profiles and the number of aeroallergen sensitization categories could be explored in the archived dataset, the timing of serum sampling was available only as broad spring–summer versus autumn–winter periods rather than as allergen-specific pollen exposure windows. Consequently, individual samples could not be definitively classified as having been obtained in-season or off-season for each relevant pollen sensitization. In addition, the relatively small sensitization subgroups limited the statistical power of exploratory analyses according to sensitization pattern and number of sensitizations.

Another limitation is the absence of dedicated non-allergic rhinitis and local allergic rhinitis groups [44]. Inclusion of these phenotypes would have allowed a more detailed comparison of IL-17-associated inflammatory patterns across allergic and non-allergic upper-airway disease and might have provided further insight into the contribution of non-type 2/type 3 inflammatory mechanisms. Future prospective studies including allergic rhinitis, local allergic rhinitis, non-allergic rhinitis, and asthma phenotypes, with allergen-specific seasonal sampling and broader inflammatory profiling, are warranted to better characterize the role of IL-17 across the spectrum of airway inflammation.

Finally, the cross-sectional nature of the analysis precludes causal inference.

## 5. Conclusions

In conclusion, circulating serum IL-17 concentrations were significantly higher in adults with allergic rhinitis, non-allergic asthma, and allergic asthma associated with allergic rhinitis than in healthy controls. However, the substantial overlap in IL-17 distributions among the three disease phenotypes limits phenotype-specific interpretation. These findings should therefore be considered exploratory and hypothesis-generating. Further prospective studies with larger cohorts, detailed inflammatory phenotyping, and longitudinal assessment are required to determine whether circulating IL-17 has clinically relevant value within multimarker approaches to airway inflammatory endotyping.

## Figures and Tables

**Table 1:** Baseline demographic and clinical characteristics of the study population.

Characteristic	Healthy Controls (*n* = 20)	Allergic Rhinitis (*n* = 31)	Non-Allergic Asthma (*n* = 15)	Allergic Asthma + Allergic Rhinitis (*n* = 22)
Age, years, mean ± SD	25.1 ± 4.9	28.5 ± 8.2	46.2 ± 16.6	29.8 ± 12.1
Age range, years	19–35	18–52	18–65	18–57
Female sex, *n* (%)	17 (85.0)	14 (45.2)	7 (46.7)	12 (54.5)
Current smokers, *n* (%)	5 (25.0)	4 (12.9)	1 (6.7)	0 (0.0)
Positive family history of allergic disease, *n* (%)	NA	9 (29.0)	5 (33.3)	7 (31.8)
Urban residence, *n* (%)	19 (95.0)	31 (100.0)	12 (80.0)	21 (95.5)
Overweight/obesity, *n* (%)	—	11 (35.5)	11 (73.3)	9 (40.9)
Disease duration	N/A	≥2 years	≥2 years	≥2 years

Data are presented as mean ± standard deviation (SD), range, or number (%), as appropriate. NA, data not available; N/A, not applicable.

**Table 2:** Distribution of serum IL-17 concentrations across the study groups.

Group	N	Mean ± SD (pg/mL)	Median (pg/mL)	IQR (pg/mL)	Range (pg/mL)
Non-allergic asthma	15	142.44 ± 138.13	126.21	6.07–259.69	0.00–397.73
Allergic rhinitis	31	108.50 ± 124.67	50.97	0.00–222.70	0.00–344.28
Allergic asthma + allergic rhinitis	22	111.32 ± 131.24	54.72	0.00–163.53	0.00–357.74
Healthy controls	20	4.07 ± 6.69	0.00	0.00–5.86	0.00–20.70

## Data Availability

The data that support the findings of this study are available from the corresponding author upon reasonable request. The data are not publicly available because they contain clinical information and are subject to institutional and ethical restrictions.

## Abbreviations

AA + AR, allergic asthma associated with allergic rhinitis; ANOVA, analysis of variance; AR, allergic rhinitis; ARIA, Allergic Rhinitis and its Impact on Asthma; BMI, body mass index; ELISA, enzyme-linked immunosorbent assay; GINA, Global Initiative for Asthma; IL, interleukin; IQR, interquartile range; NAA, non-allergic asthma; SD, standard deviation; T2, type 2; Th17, T helper 17.
